# 819. Exploring Vitamin D’s Role in Wound Healing and Psychiatric Morbidities Following Deep Extremity Burns

**DOI:** 10.1093/jbcr/irag033.258

**Published:** 2026-04-06

**Authors:** Paul Won, Matthew Q Dao, Sarah Wang, Paige Baranco, Eloise W Stanton, Paige K Zachary, Sarah A Stoycos, T Justin Gillenwater, Haig A Yenikomshian

**Affiliations:** Division of Plastic Surgery, Department of Surgery, McGovern Medical School, University of Texas Health Science Center at Houston, Houston, TX; University of Texas Medical Branch - John Sealy School of Medicine, Galveston, TX; Keck School of Medicine, University of Southern California, Los Angeles, CA; Los Angeles Medical Center, Los Angeles, CA; Division of Plastic and Reconstructive Surgery, Keck School of Medicine, University of Southern California, Los Angeles, CA; Division of Plastic and Reconstructive Surgery, Keck School of Medicine, University of Southern California, Los Angeles, CA; Keck School of Medicine, University of Southern California, Los Angeles, CA; Division of Plastic and Reconstructive Surgery, Keck School of Medicine, University of Southern California, Los Angeles, CA; Division of Plastic and Reconstructive Surgery, Keck School of Medicine, University of Southern California, Los Angeles, CA

## Abstract

**Introduction:**

Vitamin D is essential for immune regulation, collagen remodeling, and musculoskeletal recovery, which are foundational in wound healing. Beyond physical recovery, vitamin D also influences neuropsychiatric function via neurotransmitter regulation. Although vitamin D deficiency is prevalent in burn patients, there remains a paucity of large-scale data on its physical and psychological impacts. This study evaluated post-burn vitamin D deficiency and its association with wound complications as well as psychiatric disorders following extremity burns.

**Methods:**

Using the US Collaborative Network in the TriNetX database of anonymized medical records, patients (≥18 years) with second- or third-degree extremity burns were identified. Patients were then stratified by vitamin D status, with deficiency defined as 25(OH)D3 < 20 ng/mL after burn injury. 1:1 propensity score matching was done for demographics (e.g., age, race, ethnicity, socioeconomic status, and sex), body mass index (BMI), comorbidities (e.g., diabetes, chronic kidney and liver disease, cardiovascular disease, and pancreatitis), burn severity (e.g., total body surface area affected and depth), and procedural treatment (debridement and skin grafting). Primary outcomes analyzed wound healing and functional complications, while secondary outcomes assessed the onset of psychiatric disorders. Adjusted odds ratios (aOR) were calculated to compare outcomes within the span of one year after burn injury. Statistical significance was defined as *p*<.05.

**Results:**

After 1:1 propensity matching, 13 391 patients remained in each cohort. Vitamin D-deficient patients and control patients were similar in average age (53.4 vs 54.6 years) and BMI (30.4 vs 30.5 kg/m^2^). Matched patients with vitamin D deficiency had significantly higher odds of hypertrophic scar formation (aOR: 1.62, *p*<.001), wound infection (aOR: 1.51, *p*=.003), wound dehiscence (aOR: 1.54, *p*=.001), joint (aOR: 1.67, *p*<.0001) and muscle contractures (aOR: 1.61, *p*=.010), postoperative pain (aOR: 1.46, *p*<.001), muscle atrophy (aOR: 2.58, *p*<.001), and emergency department visits (aOR: 1.21, *p*=.009) compared to matched controls. Additionally, higher odds of depression (aOR: 1.68, *p*<.001), anxiety (aOR: 1.60, *p*<.001), and opioid use disorder (aOR: 1.34, *p*=.027) were observed in the matched vitamin D-deficient patients compared to matched controls.

**Conclusions:**

Vitamin D deficiency following deep extremity burns is linked to greater rates of wound complications, functional impairments, and psychiatric disorders. These findings support vitamin D supplementation after burn injury as a necessary and important factor for recovery.

**Applicability of Research to Practice:**

Routine screening and treatment of vitamin D deficiency may improve wound healing and psychiatric health in burn patients. Prospective research is warranted to assess the efficacy of vitamin D supplementation as part of standard burn care protocols.

**Funding for the study:**

N/A.

